# Farming on Mars: Treatment of basaltic regolith soil and briny water simulants sustains plant growth

**DOI:** 10.1371/journal.pone.0272209

**Published:** 2022-08-17

**Authors:** Pooja Kasiviswanathan, Elizabeth D. Swanner, Larry J. Halverson, Paramasivan Vijayapalani

**Affiliations:** 1 Ames High, Ames, Iowa, United States of America; 2 Department of Geological & Atmospheric Sciences, Ames, Iowa, United States of America; 3 Department of Plant Pathology & Microbiology, Iowa State University, Ames, Iowa, United States of America; Free University of Bozen-Bolzano, ITALY

## Abstract

A fundamental challenge in human missions to Mars is producing consumable foods efficiently with the *in situ* resources such as soil, water, nutrients and solar radiation available on Mars. The low nutrient content of martian soil and high salinity of water render them unfit for direct use for propagating food crops on Mars. It is therefore essential to develop strategies to enhance nutrient content in Mars soil and to desalinate briny water for long-term missions on Mars. We report simple and efficient strategies for treating basaltic regolith simulant soil and briny water simulant for suitable resources for growing plants. We show that alfalfa plants grow well in a nutrient-limited basaltic regolith simulant soil and that the alfalfa biomass can be used as a biofertilizer to sustain growth and production of turnip, radish and lettuce in the basaltic regolith simulant soil. Moreover, we show that marine cyanobacterium *Synechococcus sp*. PCC 7002 effectively desalinates the briny water simulant, and that desalination can be further enhanced by filtration through basalt-type volcanic rocks. Our findings indicate that it is possible to grow food crops with alfalfa treated basaltic regolith martian soil as a substratum watered with biodesalinated water.

## Introduction

Human exploration of Mars is a future mission goal for the U.S. National Aeronautics and Space Administration, and is driving technology development to support such an endeavor. While the vision to sustain human activity on Mars is compelling, providing consumables to the crews is a fundamental challenge due to transport costs and need for continuous replenishment. Consequently, long-term human missions to Mars will rely on using martian resources available *in situ* to develop food production system. Yet, many resources such as soil, water, solar radiation, carbon and nitrogen found on the martian surface [1] cannot be directly exploited by plants for growth in the form which they are found.

Mars’ surface is mostly basaltic in composition as a consequence of past volcanism [2–4]. Martian regolith soil is largely weathered basalt, which contains salts such as sulfates and perchlorates [4]. Analyses have shown that basaltic regolith soil contain basic macro elements (C, H, O, N, P, S, K, Mg, Na and Ca) and minor elements (Mn, Cr, Ni, Mo, Cu, Fe and Zn) essential for life [5–7]. However, the main limiting factors of the basaltic regolith soil are its low nutrient bioavailability and poor water-holding capacity due to absence of organic carbon [8–10]. Most surface water on present Mars is in the form of polar ice sheets, and subglacial liquid has been detected in these areas [11,12]. Several lines of evidence also suggest the occurrence of deliquescence processes caused by hygroscopic salts or by permafrost melts resulting in the formation of liquid brines or saline seeps [13–17]. Due to the geochemical properties of martian basaltic regolith soil and briny water, these *in situ* resources are unsuitable for growing plants for food. Hence, there is a critical need for suitable resources for growing plants on Mars.

A first step towards enabling habitation of Mars is the optimization of *in situ* resources as suitable resources for growing plants as part of a bioregenerative food system [18]. Consumable plants are an essential part of bioregenerative systems but also have secondary benefits such as contributing to generation of oxygen and organic waste recycling [19,20]. Although there is no location on Earth exactly reproducing martian environmental conditions, the harsh martian climate and geological features are comparable to some places on Earth [21–23]. The use of terrestrial analogues has therefore lead to the understanding of the physical, geochemical and environmental conditions that occur on Mars and help to resolve limitations to habitation relying on martian resources [8,24–33]. Microbes and higher plants have the potential to adapt to extreme terrestrial environments that simulate martian conditions [7,8,10,24,25,27,34–37]. Photosynthetic bacteria that are commonly employed in seawater biodesalination technologies allow cost and energy-efficient desalination [38,39]. Exploitation of such microbes in desalination of martian briny water may provide a usable water resource. Application of such potential biologicals in treating basaltic regolith soil and briny water for suitable resources for raising food crops would benefit future Mars missions. Here, we aimed to find strategies for treating basaltic regolith simulant soil and briny water simulant as suitable resources for growing food crops.

## Materials and methods

### Basaltic regolith soil simulant

Natural basalt-type volcanic rocks (Pleasant Hearth and Ace Hardware Corporation) were crushed in a Bico pulverizer (Department of Geological and Atmospheric Sciences, Iowa State University) to particles of 3 mm diameter or smaller and used as basaltic regolith simulant soil. Element composition and physical properties of the simulant soil were analyzed by Solum Inc. (Ames, Iowa, USA). The simulant soil was subjected to Mehlich 3 extraction [40] and mineral element content was analyzed by inductively coupled plasma spectrometry. A continuous flow system (Timberline TL 2900) was used for analyzing nitrogen content according to the manufacturer’s instructions. Water retention capacity of the simulant soil was determined from difference between constant weight of water saturated soil and dried soil. Two weeks after seed germination in basaltic regolith simulant soil, the soil (100 g) was treated with 15 ml chemical fertilizer (16.55% total nitrogen, 4.93% phosphate, 16.29% potash, 1.51% magnesium, 0.016% boron, 0.007% copper, 0.082% iron, 0.04% manganese, 0.007% molybdenum and 0.04% zinc; Jack’s Professional LX) or 100 mg of powder of alfalfa (grown in bare basaltic regolith soil) on a weekly basis. As plant species enriched in macronutrients, micronutrients, minerals, vitamins, amino acids and hormones could potentially be used as fertilizers in soil management [41], we assessed growth of alfalfa in bare basaltic regolith simulant soil and used powder of those alfalfa plants to treat the basaltic regolith soil. Garden soil was included as a control. In each plant growth experiment, at least 5 pots per soil type were used as replicates.

### Briny water simulant

BG-11 medium containing a mixture of inorganic salts was prepared as described in the University of Texas Algal Culture Collection (UTCC) recipe [42]. Sodium chloride was added to the BG-11 medium to 0.343 mol L^-1^ (briny water simulant) and autoclaved at 121°C and 15 psi for 20 min. Salinity (32.5 practical salinity unit, PSU) and electrical conductivity (EC; 48 mS/cm) of the briny water simulant were determined using a Thermo Electron-Orion Conductivity Benchtop Meter.

### Growth of *Synechococcus* sp. strain PCC 7002 in briny water simulant

Marine cyanobacterium *Synechococcus* sp. PCC 7002 (*Agmenellum quadruplicatum*) desalinates high salinity water [39] and we tested the efficacy of *Synechococcus* sp. PCC 7002 to desalinate the briny water simulant. *Synechococcus sp*. PCC 7002 was obtained from the UTCC and maintained on BG-11 agar medium containing 0.171 mol L^-1^ NaCl and 1.5% agar. To the autoclaved BG-11 agar medium, 1 mmol L^-1^ sodium thiosulphate pentahydrate and 0.1% vitamin B12 in 50 mmol L^-1^ HEPES buffer (pH 7.8) were added prior to use. A dense lawn of *Synechococcus* sp. PCC 7002 was inoculated into a 250 ml Erlenmeyer flask containing 100 ml of briny water simulant and grown statically in a growth chamber (Percival-Scientific) under white soft light ≤650 μmol photons/m^2^/s for 16 h at 26°C and dark for 8 h at 24°C. Growth of *Synechococcus* sp. PCC 7002 in the briny water simulant over time was determined by measuring absorbance of the culture at 730 nm in a Beckman spectrophotometer. Salinity and EC of the briny water simulant were measured during *Synechococcus* sp. PCC 7002 growth.

### Filtration of *Synechococcus* sp. PCC 7002 grown in briny water simulant

**Fig 1:**
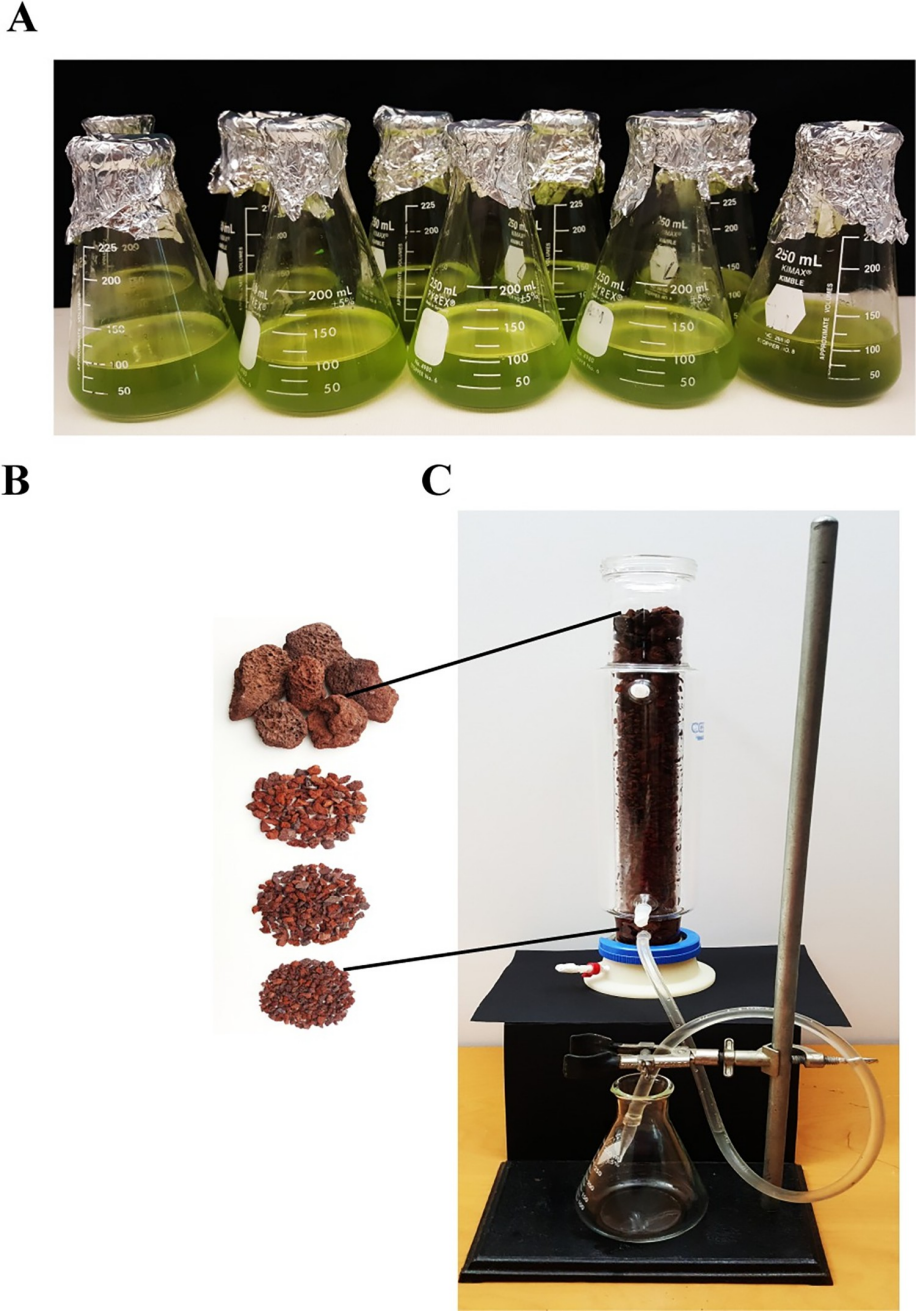
Gravity filtration of *Synechococcus* sp. PCC 7002 cultured in briny water simulant through basalt-type volcanic rocks. (A) Flasks containing *Synechococcus* sp. PCC 7002 grown briny water simulant. (B and C) Image of basalt-type volcanic rocks packed in a glass column with largest particles (~60 mm) at the top, smallest particles (~ 6 mm) at the bottom and intermediate particles in between. Final filtrate was collected and used to grow plants.

*Synechococcus* sp. PCC 7002 cultured briny water simulant (100 ml) (Fig 1A) was filtered by gravitational flow through a bed of basalt-type volcanic rocks packed tightly in a 1 L Pyrex glass column (Fig 1B and 1C). Filtrate was collected and re-filtered twice in the same column. Salinity and EC were measured on the final filtrate.

### Plant growth experiments

Turnip (*Brassica rapa*), radish (*Raphanus sativus*), lettuce (*Lactuca sativa*) or alfalfa (*Medicago sativa*) seeds were sown in pots containing basaltic regolith simulant soil or garden soil and germination was assessed after a week. Plants were grown in a growth chamber (Percival-Scientific) under controlled condition [16 h white soft light (650 μmol photons/m^2^/s) at 26°C and 8 h dark at 24°C] and watered once a week. Plant shoot height and root length were measured at 1 week interval. To determine biomass, individual (whole) plants were dried at 37°C in an incubator until it reached a constant weight and dry weight was measured, or fresh weight of individual plant bulbs was measured. Alfalfa was grown in bare basaltic regolith simulant soil, dried at room temperature and hand crushed into powder.

In each experiment, at least three replicates were used. Data represent average ± standard error (SE). Significance between treatments were statistically analyzed by paired *t*-test or one-way ANOVA with JMP16 (SAS Institute, NC, USA) and post-hoc tests were determined by Tuckey’s HSD.

## Results

### Seed germination and plant growth in basaltic regolith simulant soil

Analysis of basaltic regolith simulant soil showed a low content of nitrate, ammonium, phosphorous, potassium, sulfur, zinc, manganese, boron, calcium, magnesium, iron and copper relative to garden soil (Table 1). The simulant soil was poor in organic matter content and cation exchange capacity, and alkaline as compared to garden soil.

**Table 1:** Properties of basaltic regolith simulant soil.

Property	Basaltic regolith simulant soil	Garden soil*
NO3-N (mg g^-1^)	0.001	0.0174
NH4-N (mg g^-1^)	0.0002	0.0037
Mineral element (mg g^-1^) P K S Zn Mn B Ca Mg Fe Cu	0.0177 0.0845 0.0022 0.0007 0.019 0.0006 0.4756 0.1565 0.4519 0.0033	0.0911 0.4463 0.01 0.0054 0.0552 0.0019 3.625 0.5651 0.5651 0.0024
Organic matter (% mass)	0.2	6.9
Cation exchange capacity (meq/100g)	4.0	21.8
pH	8.6	7.0

*control.

**Fig 2:**
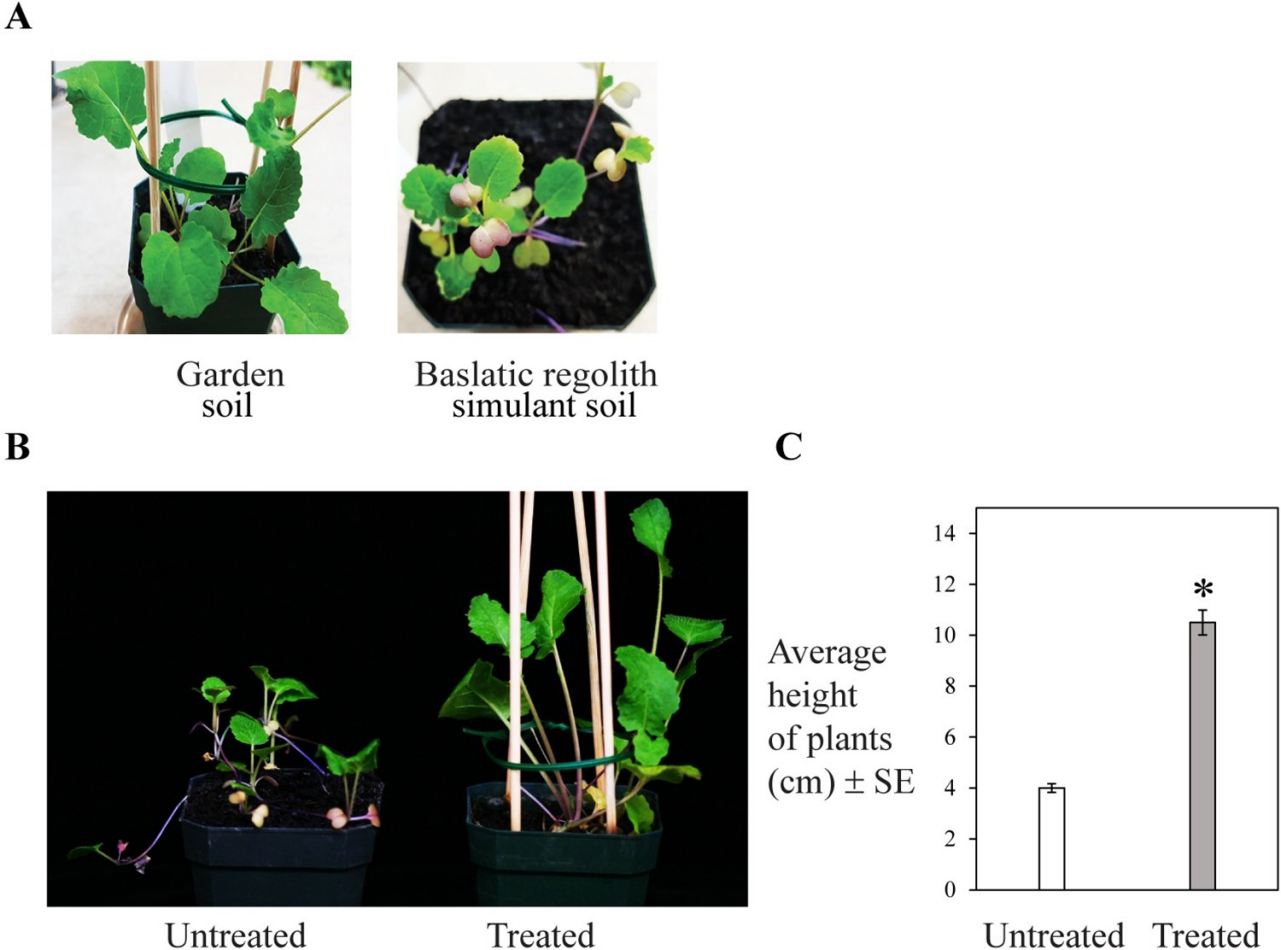
(A) Growth of turnip plants (after 3 weeks) in basaltic regolith simulant soil or garden soil watered with fresh water. (B and C) Growth of turnip plants (after 4 weeks) in chemical fertilizer treated or untreated basaltic regolith simulant soil watered with fresh water. Shoot height of at least 5 individual plants in each treatment was measured. Data represent average ± SE and significant difference is indicated by an asterisk (p < 0.01) as determined by paired *t*-test.

Turnip seed germination in basaltic regolith simulant soil and garden soil (watered with fresh water) was compared. In basaltic regolith simulant soil, 62% of the seeds germinated within a week, while 55% of the seeds germinated in garden soil. Turnip plants grown in basaltic regolith simulant soil were stunted and produced fewer and smaller discolored leaves than plants grown in garden soil (Fig 2A**)**. Overall, the growth of turnip plants in the basaltic regolith simulant soil was unhealthy as compared to that grown in garden soil.

Growth of turnip plants in basaltic regolith simulant soil was then assessed after a chemical fertilizer treatment. Shoot height of turnip plants in fertilizer treated simulant soil was significantly increased (163%) relative to plants in unfertilized simulant soil (Fig 2B and 2C). Chemical fertilization of the simulant soil resulted in improvement in overall growth and normal phenotype of the turnip plants.

### Growth of food crops in alfalfa treated basaltic regolith simulant soil watered with fresh water

**Fig 3:**
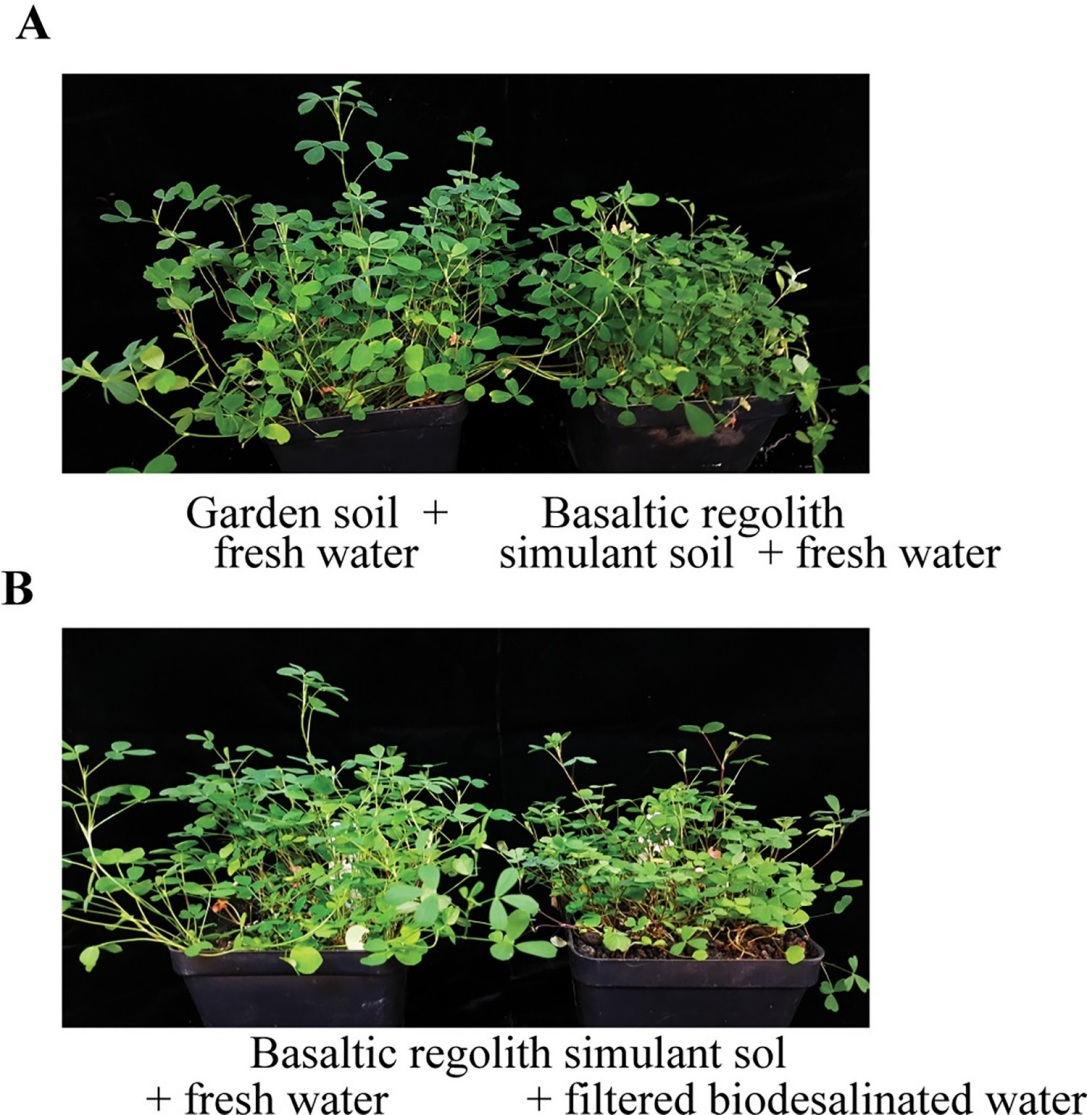
Growth of alfalfa in bare basaltic regolith simulant soil or garden soil watered with (A) fresh water or (B) fresh water or filtered biodesalinated water.

**Fig 4:**
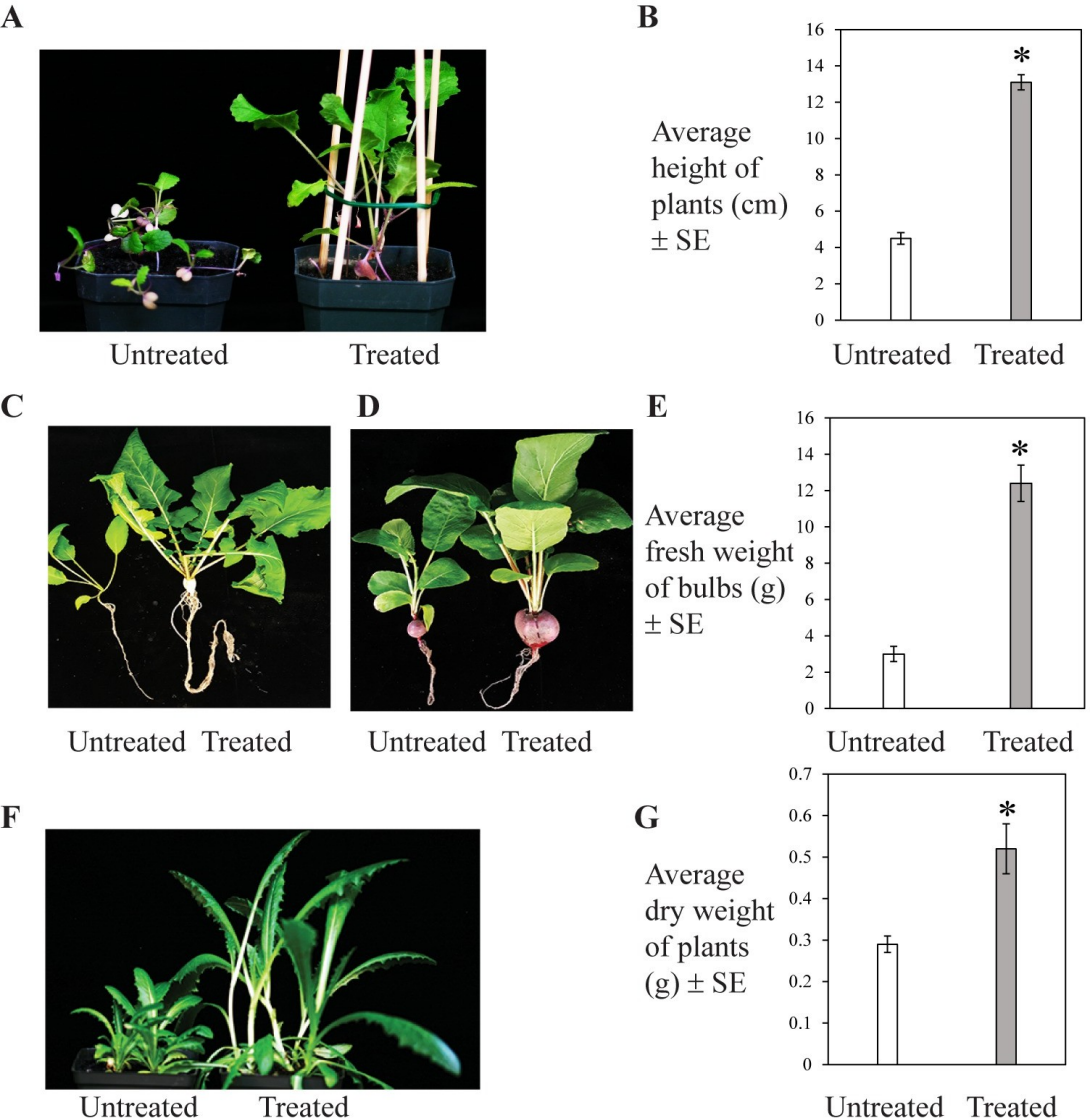
Growth of (A, B and C) turnip, (D and E) radish and (F and G) lettuce in alfalfa treated or untreated basaltic regolith simulant soil watered with fresh water. Shoot height of 5 individual turnip plants (after 4 weeks), fresh weight of 5 radish bulbs (after 7 weeks), and dry weight of 5 whole lettuce plants (after 7 weeks) per treatment were measured. Data represent average ± SE and significant difference is indicated by an asterisk (p ≤ 0.02) as determined by paired *t*-test.

As plant species enriched in macronutrients, micronutrients, minerals, vitamins, amino acids and hormones could potentially be used as fertilizers in soil management [41], in this context, we assessed growth of alfalfa in bare basaltic regolith simulant soil watered with fresh water. Given the robust growth of alfalfa in the bare simulant soil (Fig 3A), alfalfa was further tested if it can serve as a nutrient source in simulant soil for growing food crops. Growth and biomass of turnip, radish and lettuce plants in alfalfa (grown in bare simulant soil) treated simulant soil (watered with fresh water) was evaluated. Growth of all three type of plants was boosted in the alfalfa treated simulant soil as compared to that grown in untreated simulant soil (Fig 4A, 4C, 4D and 4F). Growth of turnip plants increased to 190% in alfalfa treated simulant soil (Fig 4B). Turnips plants produced healthy bulbs in alfalfa treated simulant soil (Fig 4C). Biomass of radish bulbs (311%) (Fig 4E) and lettuce leaves (79%) (Fig 4G) was significantly improved as compared to that when grown in untreated simulant soil.

### Growth of food crops in alfalfa treated basaltic regolith simulant soil watered with biodesalinated water

**Fig 5:**
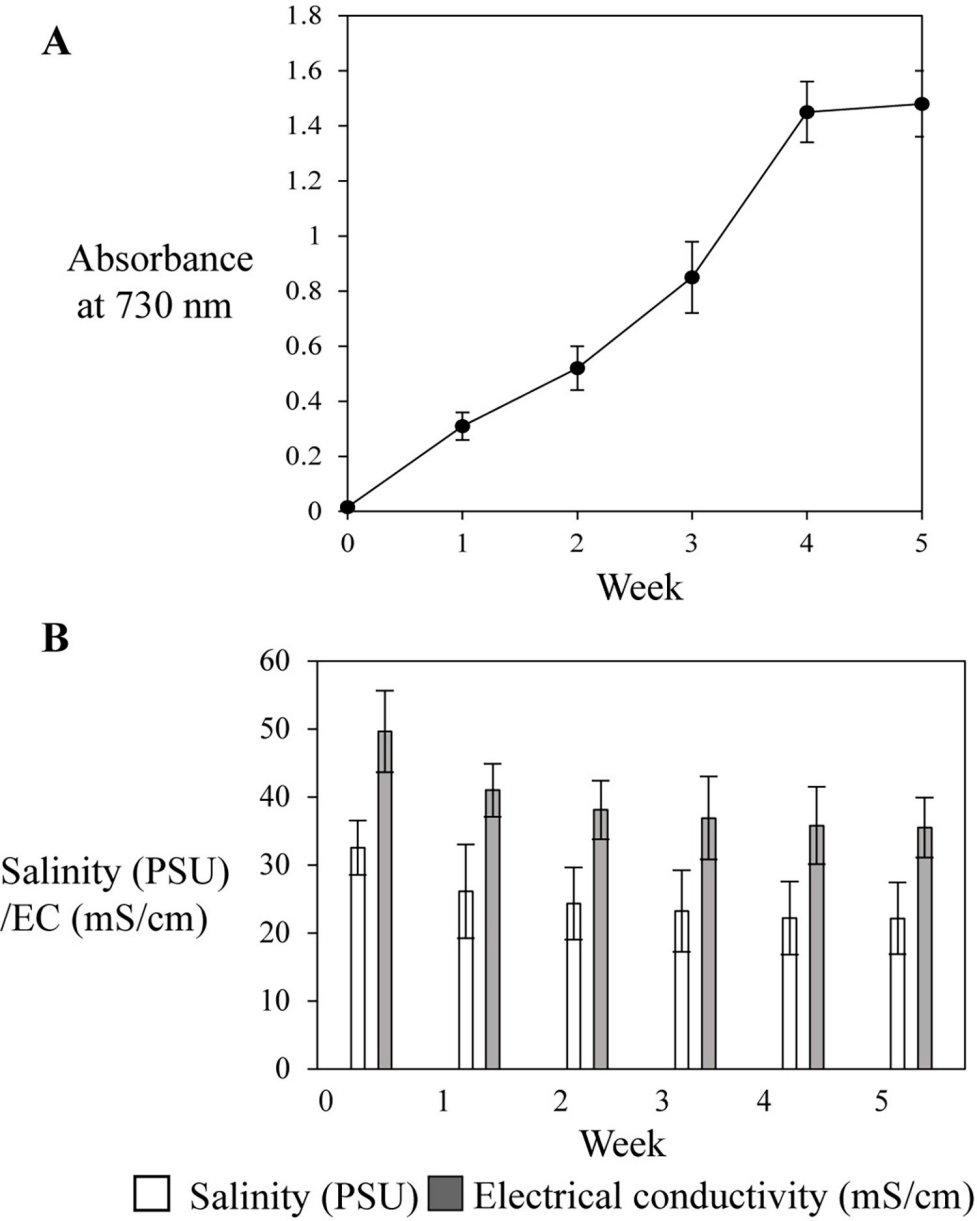
(A) Growth, (B) salinity and EC of *Synechococcus* sp. PCC 7002 inoculated briny water simulant. Measurements were made in cultures from at least 5 individual flasks. Data are the average ± SE and significant difference in salinity or EC (B) between briny water simulant starting material and simulant at each time point was analyzed by paired *t*-tests.

Absorbance of *Synechococcus* sp. PCC 7002 inoculated simulant water increased with time and growth was maximum after 4 weeks (Fig 5A). During the growth of *Synechococcus* sp. PCC 7002, salinity and EC of the simulant water gradually declined (Fig 5B). Within 4 weeks, salinity of the simulant water was reduced to about 32%, which demonstrated effective biodesalination mediated by *Synechococcus* sp. PCC 7002.

**Fig 6:**
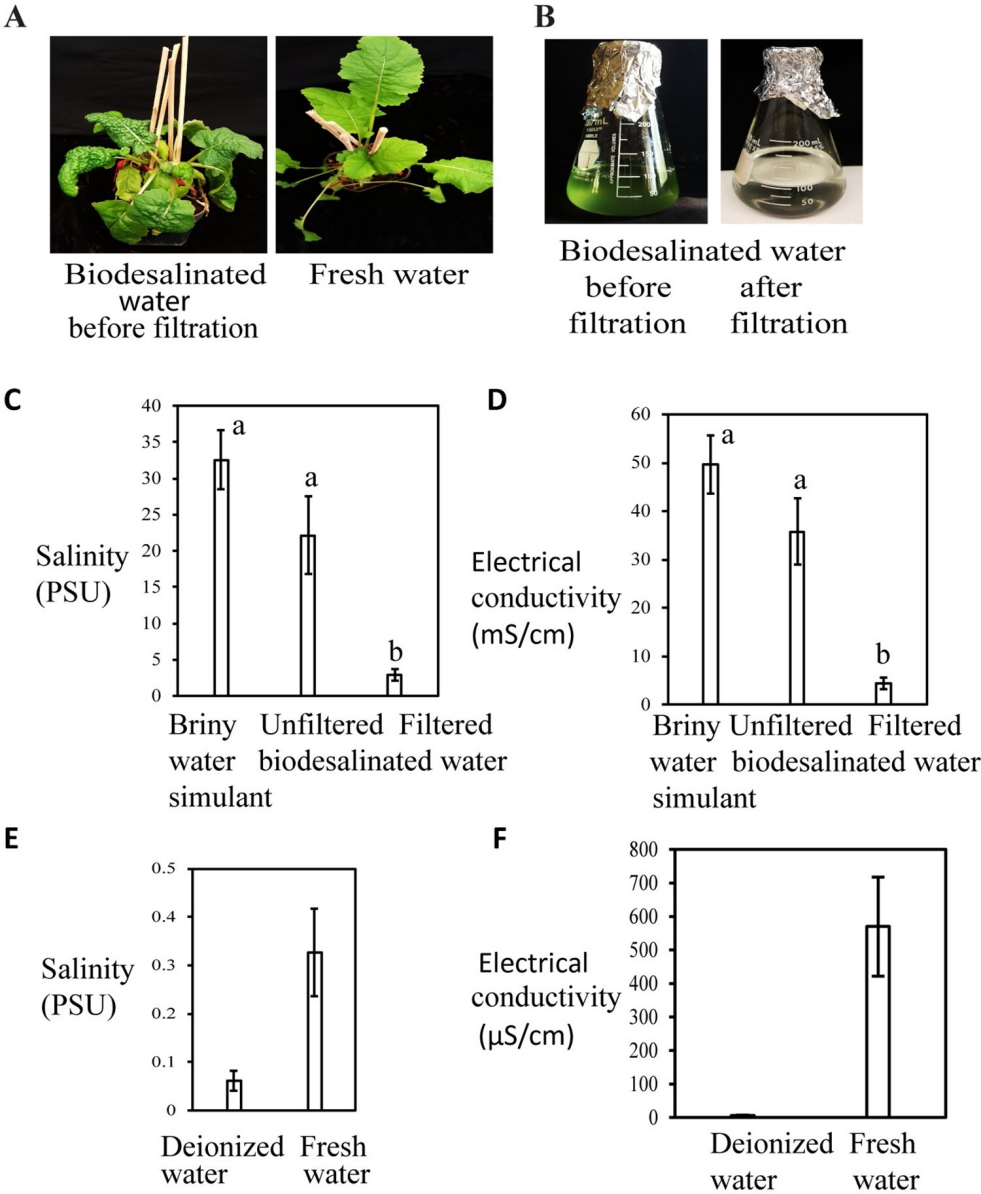
(A) Growth of turnip plants (after 3 weeks) in alfalfa treated basaltic regolith simulant soil watered with unfiltered biodesalinated water or fresh water (control). (B**)** Flasks containing biodesalinated water before or after filtration. (C) Salinity and (D) EC of unfiltered and filtered biodesalinated water, and starting briny water simulant material. (E) Salinity and (F) EC of deionized water and fresh water (controls). At least three replicates per water type were used for salinity and EC measurements. Data represent average ± SE. (C and D) Average salinity or EC of samples that do not share a letter above the bar are significantly different (P<0.0001) as determined by one-way ANOVA.

We then assessed growth of turnip plants in alfalfa treated basaltic regolith simulant soil watered with biodesdalinated water. Turnip plants did not produce healthy shoots when watered with biodesalinated briny water as compared to that grown with fresh water (Fig 6A). Following these results, *Synechococcus* sp. PCC 7002 cells grown in the biodesalinated water were removed by filtration through basalt-type volcanic rocks. Absorbance of the final filtrate was reduced to 0.25 (initial absorbance, 1.45) (Fig 6B). Both salinity and EC of the final filtrate was significantly reduced to 91%, as compared to the starting material (Fig 6C and 6D).

**Fig 7:**
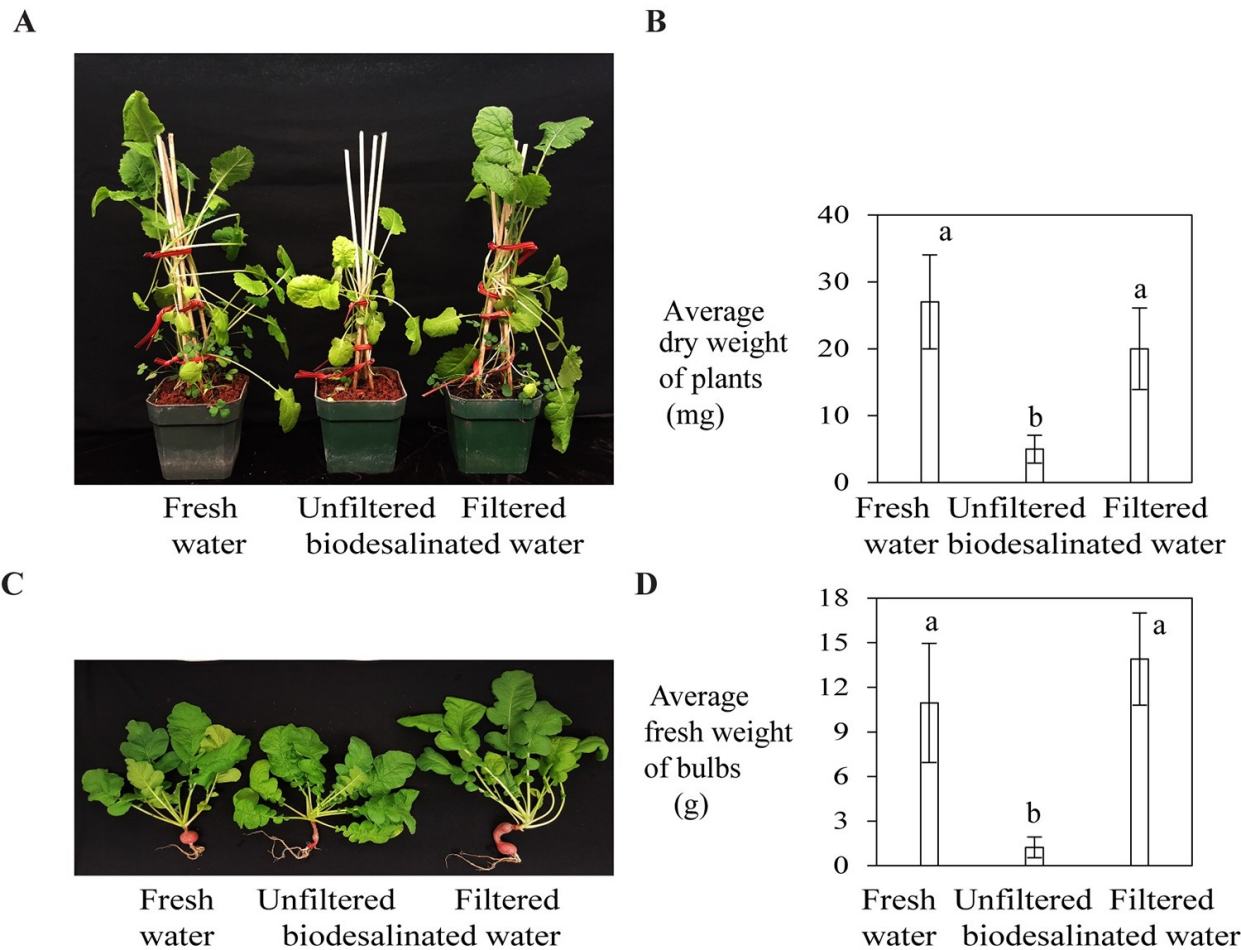
Growth of (A and B) turnip and (C and D) radish plants (after 6 weeks) in alfalfa treated basaltic regolith simulant soil watered with filtered or unfiltered biodesalinated water, or fresh water (control). Dry weight of 5 whole turnip plants and fresh weight of 5 radish bulbs in each treatment type were measured. Data represent average ± SE. (B and D) Average dry or fresh weight of samples that do not share a letter above the bar are significantly different (P<0.0001) as determined by one-way ANOVA.

Effect of filtered biodesalinated water on growth of turnip and radish plants was further evaluated in alfalfa treated basaltic regolith simulant soil. Both turnip and radish plants grew healthily with filtered biodesalinated water as compared to plants grown with unfiltered biodesalinated water (Fig 7A and 7C). There was a significant increase in dry weight of turnip plants (278%) and fresh weight of radish bulbs (1047%) grown with filtered biodesalinated water relative to that of plants grown with unfiltered biodesalinated water (Fig 7B and 7D).

## Discussion

One of the grand challenges of future human missions to Mars is the complexity of sending the required consumables from Earth. An alternative approach is to produce consumables utilizing martian *in situ* resources [7]. The geochemical properties of martian basaltic regolith soil and briny water require strategies for treating these resources as suitable resources, that may lead to production of consumable plants [7,18]. In mineral composition, Mars regolith simulants are comparable to Earth soils [43,44] and they can be mimicked by using basalt [10,45]. Mars regolith analogs have been tested for growing plants and established that the capacity of specific regolith simulants supported plant growth only in short term without nutrient supplementation [10,18,28,31,32,45–47]. We aimed to find strategies for treating basaltic regolith simulant soil and briny water simulant as suitable resources for sustainable growth of food crops. For this, we used a basaltic regolith simulant soil that was poor in nutrient contents (Table 1) and aimed to grow a plant species that could grow in the simulant soil without supplementation of nutrients and to use those plants as fertilizer for growing crop plants in the bare simulant soil.

Before testing basaltic regolith simulant soil for plant growth, the soil was sterilized in an autoclave. Turnip plants grown in the basaltic regolith simulant soil watered with fresh water grew slowly and had an unhealthy phenotype. The reversal of unhealthy to healthy turnip plants in the basaltic regolith simulant soil after a chemical fertilizer treatment confirmed the need to augment the simulant soil with nutrients for normal plant growth. In this context, alfalfa was explored as a fertilizer of the basaltic regolith simulant soil. Alfalfa could grow in the bare basaltic simulant soil without fertilizer and the growth of alfalfa was comparable in bare basaltic regolith simulant soil and garden soil. Alfalfa was chosen as an ideal biofertilizer since: i) this species has tiny seeds so that the nutrient stock in the seeds would be quickly depleted and the plant becomes totally dependent on what is available in the bare simulant soil for growth and ii) if alfalfa could grow in basaltic regolith simulant soil that is naturally poor in nutrients, then after growth, alfalfa can be mixed with the simulant soil as a biofertilizer to augment nutrients.

Turnip, radish and lettuce plants have a very high harvest index, low water uptake/transpiration ratio, brief growing cycle and require little attention [48,49]. As all these properties of these crops are desirable for consumables during martian habitation, growth of these three plant species was evaluated in alfalfa (grown in bare simulant soil as first generation biological) treated basaltic regolith simulant soil watered with fresh water. The significant increase in growth and biomass of turnip, radish and lettuce plants in alfalfa treated simulant soil demonstrated that the alfalfa grown in bare simulant soil augment nutrients to the basaltic regolith simulant soil to sustain normal growth and productivity of the next generation crops. On the other hand, the presence of perchlorate in martian regolith provides a significant challenge in its use as an agricultural substrate [50]. As we aimed to find a strategy to raise a nutrient resource (alfalfa) in the simulant itself, we did not test the effect of perchlorate on the growth of plants in the simulant soil. Our future research in this line could incorporate perchloride amendments in the simulant soil and before testing plant growth.

In alfalfa treated simulant soil watered with briny water simulant, no turnip seeds germinated within 7 days of sowing, while 100% of the seeds germinated in 3 days when watered with fresh water. Due to the inhibitory effect of high salinity of briny water simulant water on seed germination, we aimed to desalinate the simulant water. Desalination strategies such as reverse osmosis typically require synthetic membranes, and high electrical energy consumption has limited their application [51–54]. Alternatively, utilization of microbes for desalination could be cost-effective and energy-efficient [38]. Bacterial species grow in extremely high concentrations of solutes including saturated NaCl and MgSO_4_ [55,56]. Halobacteria from saline lakes (considered as analogues of ancient martian organisms) withstand low temperatures and high NaCl content similar to conditions in Mars [57]. Terrestrial microbes including fungus grow in densest brines such as those on Mars [16,17]. Considering the potential adaptation of terrestrial microbes to extreme salinity as found in Mars, we explored the use of microbes to desalinate the briny water simulant.

Autotrophic cyanobacteria of marine origin can bloom in seawater with minimal nutrient requirements and under natural sunlight. The ease of growth and maintenance favor the use of planktonic cultures of cyanobacteria in biodesalination [58–61]. *Synechococcus* sp. PCC 7002 can desalinate high salinity water [39], and we tested the efficacy of *Synechococcus* sp. PCC 7002 to desalinate the briny water simulant. Growth of *Synechococcus* sp. PCC 7002 in briny water simulant led to 32% reduction in salinity. *Synechococcus* sp. PCC 7002 mediated biodesalinated water did not support normal growth of turnip plants in alfalfa augmented basaltic regolith simulant soil. We then attempted to eliminate the intact cyanobacterial cells from the biodesalinated water in order to preclude inadvertent release of sodium chloride from the cells. The challenge in separating planktonic cyanobacterial cells from aqueous suspensions stems from the fact that cells have similar densities to water, cells behave like colloidal particles, and cells possess charged surfaces that stabilize cell suspensions [38]. A simple gravity filtration for *Synechococcus* sp. PCC 7002 biomass removal was attempted using the naturally porous basalt-type volcanic rocks. The filtration process resulted in a remarkable removal of PCC 7002 biomass, as evident from the reduction in absorbance (83%) of the final filtrate. Most importantly, the rock filtration further lead to a 91% reduction in salinity, which could be accounted for by adsorption of inorganic salts onto the basalt-type volcanic rocks. Application of the filtered biodesalinated water sustained normal growth and biomass production of turnip and radish plants in alfalfa augmented basaltic regolith simulant soil. Together, the results indicate that the biodesalination of briny water simulant mediated by *Synechococcus* sp. PCC 7002 combined with rock filtration is a simple and effective desalination strategy, that could be scaled-up.

We report simple and efficient strategies for treating basaltic regolith soil and briny water simulants, and demonstrate that the treated simulants can sustain normal growth of food crops. Collectively, the efficient growth of the three plant species in the alfalfa augmented basaltic regolith simulant soil with *Synechococcus* sp. PCC 7002 mediated biodesalinated water supports, that in principle, it is possible to grow food crops in treated martian basaltic regolith soil watered with biodesalinated water. A next step would be to test growth of cereal and leguminous crops in the treated simulant soil. Alfalfa and *Synechococcus* sp. PCC 7002 thus served as first generation life supporting biologicals for treating the basaltic regolith simulant soil and briny water simulant, respectively, for suitable resources for growing plants. For the first time, we report an integrated use of a biofertilizer and microbe for effective treatment of basaltic regolith soil and briny water simulants, respectively, for suitable resources that sustain plant growth. Furthermore, this study signifies that for long-term purposes, it is possible to treat *in situ* soil and water resources for farming on Mars to sustain human missions and permanent settlements.

## Supporting information

S1 Table Raw data of growth of turnip plants in chemical fertilizer treated or untreated basaltic regolith simulant soil watered with fresh water in Fig 2C in the manuscript. (XLSX)

S2 Table Raw data of growth of turnip (B), radish (E) and lettuce (G) in alfalfa treated or untreated basaltic regolith simulant soil watered with fresh water in Fig 4 in the manuscript. (XLSX)

S3 Table Raw data of growth (A), salinity and EC (B) of *Synechococcus* sp. PCC 7002 inoculated briny water simulant in Fig 5 in the manuscript. (XLSX)

S4 Table Raw data of Salinity (C) and EC (D) of unfiltered and filtered biodesalinated water, and starting briny water simulant material, and salinity (E) and EC (F) of deionized water and fresh water (controls) in Fig 6 in the manuscript. (XLSX)

S5 Table Raw data of growth of turnip (B) and radish (D) plants in alfalfa treated basaltic regolith simulant soil watered with filtered or unfiltered biodesalinated water, or fresh water (control) in Fig 7 in the manuscript. (XLSX)
